# Cyclic electron flow and light partitioning between the two photosystems in leaves of plants with different functional types

**DOI:** 10.1007/s11120-019-00666-1

**Published:** 2019-09-13

**Authors:** Julius Ver Sagun, Murray R. Badger, Wah Soon Chow, Oula Ghannoum

**Affiliations:** 1 ARC Centre of Excellence for Translational Photosynthesis, Hawkesbury Institute for the Environment, Western Sydney University, Hawkesbury Campus, Locked Bag 1797, Penrith, NSW 2751 Australia; 2 ARC Centre of Excellence for Translational Photosynthesis, Research School of Biology, Australian National University, Canberra, ACT 2601 Australia

**Keywords:** C4 photosynthesis, Chlorophyll fluorescence, Cyclic electron flux, Electron transport rate, Oxygen exchange rate, Photosystem

## Abstract

Cyclic electron flow (CEF) around photosystem I (PSI) is essential for generating additional ATP and enhancing efficient photosynthesis. Accurate estimation of CEF requires knowledge of the fractions of absorbed light by PSI (*f*_I_*)* and PSII (*f*_II_), which are only known for a few model species such as spinach. No measures of *f*_I_ are available for C_4_ grasses under different irradiances. We developed a new method to estimate (1) *f*_II_ in vivo by concurrently measuring linear electron flux through both photosystems $\left( {{\text{LEF}}_{{{\text{O}}_{ 2} }} } \right)$ in leaf using membrane inlet mass spectrometry (MIMS) and total electron flux through PSII (ETR2) using chlorophyll fluorescence by a Dual-PAM at low light and (2) CEF as ETR1—${\text{LEF}}_{{{\text{O}}_{ 2} }}$. For a C_3_ grass, *f*_I_ was 0.5 and 0.4 under control (high light) and shade conditions, respectively. C_4_ species belonging to NADP-ME and NAD-ME subtypes had *f*_I_ of 0.6 and PCK subtype had 0.5 under control. All shade-grown C_4_ species had *f*_I_ of 0.6 except for NADP-ME grass which had 0.7. It was also observed that *f*_I_ ranged between 0.3 and 0.5 for gymnosperm, liverwort and fern species. CEF increased with irradiance and was induced at lower irradiances in C_4_ grasses and fern relative to other species. CEF was greater in shade-grown plants relative to control plants except for C_4_ NADP-ME species. Our study reveals a range of CEF and *f*_I_ values in different plant functional groups. This variation must be taken into account for improved photosynthetic calculations and modelling.

**Electronic supplementary material:**

The online version of this article (10.1007/s11120-019-00666-1) contains supplementary material, which is available to authorized users.

## Introduction

Photosynthetic electron transport in the thylakoid membrane of chloroplasts is highly regulated to cope with fluctuating light intensity and variable demand for ATP and NADPH. Photon energy absorbed by pigments and the light-harvesting complexes drives electron transport through the thylakoid membranes. Electrons produced from the splitting of water molecule in photosystem II (PSII) are ultimately transferred via the cytochrome cyt *b*_6_/*f* complex and photosystem I (PSI) to NADP^+^, resulting in the production of reducing equivalents in the form of NADPH. These two processes, known as linear electron flow (LEF), generate a proton gradient across the thylakoid membrane (∆pH). The ∆pH together with a membrane potential formed across the thylakoid membrane (∆*ψ*) drives the production of ATP via ATP synthase (Allen 2003).

During cyclic electron flow (CEF), NADPH or ferredoxin (Fd) is photoreduced at PSI and donates electrons to the cyt *b*_6_/*f* complex via the plastoquinone (PQ) pool. There, the Q-cycle transfers 1 H^+^ from the stroma to the lumen for each electron donated, resulting in a ∆pH, which can drive ATP synthesis without producing NADPH in chloroplasts (Allen 2003). This process is not only key to photo-protection, but also essential for increasing the ATP/NADPH ratio. Depending on the environmental and/or physiological conditions, this ratio can be adjusted to the required levels by tuning the ratio of LEF to CEF (Miyake 2010; Shikanai 2007; Takahashi and Badger 2011).

In angiosperms, CEF operates through two known pathways (Yamori and Shikanai 2016). The major pathway depends on two additional proteins, PROTONGRADIENT REGULATION 5 (PGR5) (Munekage et al. 2002) and PGR5-LIKE PHOTOSYNTHETIC PHENOTYPE 1 (PGRL1) (DalCorso et al. 2008), whereas the minor activity pathway is mediated by a chloroplast NADH dehydrogenase-like (NDH) complex (Burrows et al. 1998; Horváth et al. 2000; Shikanai et al. 1998). Antimycin A is an inhibitor of PGR5/PGRL1-dependent CEF, but the site of inhibition has long been unclear in chloroplasts (Munekage et al. 2002).

In the absence of PGR5/PGRL1-dependent pathway, the chloroplast NDH-dependent pathway compensates for the loss of the important pathway to some extent (Munekage et al. 2004; Shikanai 2014). The chloroplast NDH complex, which is insensitive to antimycin A, recycles electrons from ferredoxin to plastoquinone and subsequently to PSI through the cyt *b*_6_/*f* complex (Shikanai 2016). In contrast to higher plants, Godde (1982) showed that the green alga *Chlamydomonas reinhardtii* CW-15 was able to use NADH as electron donor for its photosynthetic electron flow. They also showed that NDH is sensitive to rotenone and thenoyltrifluoroacetone (TTFA). This finding is important because it was recently shown that the NDH system is the main pathway for CEF in Paniceae C_3_ and C_4_ grasses (Hernández-Prieto et al. 2019).

A prerequisite to understanding the role of CEF is the ability to quantify CEF under physiological conditions, which has been difficult due to the absence of a net product of CEF (Shikanai 2014). Unlike LEF, the rate of CEF cannot be monitored by O_2_ evolution or reduction of artificial electron acceptors from PSI. Fan et al. (2016) grouped current methods for measuring and inferring CEF into two categories: (1) monitor CEF directly and (2) estimate CEF from the difference between LEF through PSII (ETR2) and total flux through PSI (ETR1). They concluded that CEF quantification in C_3_ leaves is best approximated through measurements of ETR1 and ETR2 under identical conditions according to category 2.

Measurement of the electron flux through PSI (ETR1) can be done via a Y(I)-based electron flux (Klughammer and Schreiber 2008). ETR1, based on Y(I), is then calculated as

$${\text{ETR}}1 = {\text{Y}}\left( {\text{I}} \right) \times I \times 0.85 \times f_{\text{I}}$$

where *I* is the irradiance, 0.85 is the assumed leaf absorptance and *f*_I_ is the fraction of the absorbed white light partitioned to PSI. It is worth noting that it is not easy to determine *f*_I_ experimentally under variable environmental conditions; yet, calculations of ETR1 and CEF rate depend on an accurate estimation of *f*_I_. This value can be experimentally determined under low irradiance and/or in the presence of CEF inhibitors such as antimycin A, where CEF is assumed to be zero; hence, ETR2 is supposed to be approximately equal to ETR1 (*f*_I_ values are in the range 0.4–0.5) (Kou et al. 2013a, b). However, the validity of these methods needs further evaluation.

On the other hand, measurement of ETR2 on whole tissue can be better obtained by gross rate of O_2_ evolution recorded by a gas-phase oxygen electrode compared to chlorophyll fluorescence technique (Fan et al. 2016), if the latter is not optimised. Since four electrons are released for each O_2_ molecule evolved, ETR2 (now ${\text{LEF}}_{{{\text{O}}_{ 2} }}$) equals four times the gross rate of O_2_ evolution (Chow et al. 1989; Kou et al. 2013a, b). Membrane inlet mass spectrometry (MIMS), utilising the stable ^18^O_2_ isotope to differentially and simultaneously measure rates of O_2_ uptake and evolution, provides a more precise method to accurately quantify ETR2 under near-natural conditions (Beckmann et al. 2009). It also allows the CO_2_ concentration to be monitored in the cuvette to ensure photorespiration does not significantly contribute to the O_2_ uptake signal.

The overall aim of our study was to determine to what extent CEF and *f*_I_ vary between C_3_ and C_4_ plants, and in response to variation in light intensity. *f*_I_ and *f*_II_ are only known for a few species, commonly C_3_, and *f*_I_ is always assumed to be 0.5 in untested species. It is also unknown in ferns, liverwort, gymnosperms or among the various C_4_ species. In particular, C_4_ photosynthesis possesses CO_2_-concentrating mechanisms (CCM) which operates across two photosynthetic cell types (mesophyll and bundle sheath) and serves to supercharge photosynthesis and minimise photorespiration in air. C_4_ plants are broadly grouped into three biochemical subtypes according to the primary C_4_ acid decarboxylase (NADP-ME, NAD-ME and PCK) operating in the bundle sheath (Hatch 1987). Consequently, we developed a new method which can give a more reliable estimation of ETR2 based on (a) MIMS and (b) the chlorophyll fluorescence-derived Y(II) method using a Dual-PAM/F. This can concurrently measure Y(II) and ${\text{LEF}}_{{{\text{O}}_{ 2} }}$ in leaf discs in CO_2_-enriched air applied to leaf discs of C_3_ and C_4_ plants. Leaf discs from representative species of liverwort, fern and angiosperms were also measured. This method also allowed us to experimentally determine *f*_I_ and use it to calculate ETR1 and CEF in chloroplasts of all species. Measurements were taken in the presence of CEF inhibitors (antimycin A and TTFA) and/or in low irradiance where CEF is assumed to be zero. In addition, this study determined the effect of low-light (shade) condition on light partitioning between the two photosystems.

## Materials and methods

### Plant culture

Representative grass species of C_3_ (*Panicum bisulcatum*), C_4_ NADP-ME subtype (*Panicum antidotale*), C_4_ NAD-ME subtype (*Panicum miliaceum*) and C_4_ PCK subtype (*Megathyrsus maximus*) and *Zea mays* (model C_4_ NADP-ME species) were grown in vermiculite in a naturally lit greenhouse (control) made of polycarbonate at the Australian National University. The greenhouse temperature was maintained at 28/24 °C for day/night via an in-built greenhouse temperature control system. Within the greenhouse, a steel structure was placed and covered with shade cloth which was used for shade treatment. The average ambient photosynthetic photon flux densities (PPFD) and temperature during the mid-day were 800 and 300 µmol photons m^−2^ s^−1^ and 30 °C and 29 °C for control and shade treatments, respectively. Leaves were harvested from 4- to 5-week-old plants. Representative species of gymnosperms (*Ginkgo biloba* and *Wollemi nobilis*), liverwort (*Marchantia polymorpha*) and fern (*Polypodium* sp.) and spinach (model C_3_ species) grown under full sunlight were also used. All plants were watered regularly and fertilised with Osmocote® (Scotts Australia).

### Membrane inlet mass spectrometry (MIMS)

Gas exchange was measured in a closed cuvette coupled to a mass spectrometer as described by Maxwell et al. (1998) and Dual-PAM/F (Heinz Walz) (Fig. S 1). Leaf discs (1.89 cm^2^ area) were punched from the leaf and immediately placed within the chamber together with the wet filter paper supported on a mesh of equal area. The cuvette was first calibrated for oxygen and then flushed with nitrogen gas. Then, a known volume of CO_2_ was added to create an atmosphere of approximately 4% CO_2_ (high *p*CO_2_) within the chamber; ^18^O_2_ was injected to give an atmosphere of 18–21% O_2_ and the signals were allowed to stabilise for 10 min. Gas consumption and leakage from the cuvette were negligible. The leaf was then illuminated at increasing irradiance from 50 to 2000 µmol photons m^−2^ s^−1^. The chamber temperature was maintained at 28 °C.

### Measurement of ETR1

Measurement of the electron flux through PSI (ETR1) was taken via a Y(I)-based electron flux in leaf discs at 28 °C using the FIBER version of Dual-PAM (Dual-PAM/F) with a dual wavelength (830/875 nm) unit (Walz, Effeltrich, Germany) connected to the gas exchange system via a light guide to permit simultaneous measurements. The fibre optic cable was positioned within the Perspex lid at a distance of 1.0 cm from the leaf surface. The Perspex lid weakened the light intensity coming from Dual-PAM/F by ~ 87%, so we added external white actinic (AL, from a halogen lamp), strong far-red (sFR), weak far-red (wFR) and saturating light sources through various branches of the multifurcated light guide.

The photochemical yield of PSI, Y(I), in AL at a given irradiance was obtained by the percentage of the photo-oxidisable P700. The P700 redox state was measured following the method of Klughammer and Schreiber (1994). A saturation pulse (SP) (~ 10,000 µmol photons m^−2^ s^−1^), which was introduced primarily for PAM fluorescence measurement, was applied for assessment of P700 parameters. The P700 single channel in SP-analysis mode of the Dual-PAM software was used for this purpose.

The maximum photo-oxidisable P700 content (*P*_m_) was first recorded as a prerequisite for the calculation of Y(I), non-photochemical quantum yield of PSI due to donor-side limitation Y(ND) and non-photochemical quantum yield of PSI due to acceptor side limitation Y(NA). This was done by first determining a steady state by illuminating the leaf disc with wFR (~ 50 µmol photons m^−2^ s^−1^, 723 nm) for > 10 s (Fig. S 2A) coming from an external light source which was manually controlled. This intensity of wFR light was strong enough to oxidise most of the P700 in the steady state but not strong enough to drive electrons in the inter-system chain. Then, a 200-ms SP (~ 10,000 µmol photons m^−2^ s^−1^) coming from Dual-PAM/F and external light source was superimposed to photo-oxidise the remainder of the P700 (Fig. S 2A). This additional external saturating light source was connected to the TRIGGER OUT socket of Dual-PAM/F. Both pulses were triggered at the same time through the Dual-PAM software in “Trigger out” mode.

The leaf disc was light-adapted for at least 10 min with AL (1000 µmol photons m^−2^ s^−1^) to reach steady-state photosynthesis before measurements of light response curves. Light-adapted photosynthetic parameters were recorded after 8- to 10-min exposure to each AL intensity (50, 100, 200, 300, 400, 500, 750, 1000, 1500 and 2000 µmol photons m^−2^ s^−1^) and when the rate of gross oxygen evolution was stable.

Fast kinetic recording in “External trigger” mode by the Dual-PAM was first started. The leaf disc was re-illuminated with the same AL for 10 s to retain a steady state for P700^+^ measurements immediately after the photosynthetic induction step using an electronic shutter controlled by one terminal of a pulse/delay generator (Model 555, Berkeley Nucleonics, San Rafael, CA, USA) connected to Dual-PAM/F. During each 10-s illumination, at time *T *= 8.80 s (corresponding to the time point 200 ms in Fig. S 2B), data acquisition by the Dual-PAM was started by a trigger pulse from a second terminal of the pulse/delay generator. At *T *= 8.95 s, a sFR (~ 4000 µmol photons m^−2^ s^−1^) from two external light-emitting diode arrays (741 nm ± 13 nm, LED735–66–60, Roithner LaserTechnik, Vienna, Austria) was triggered on for 250 ms using a third terminal of the pulse/delay generator. The sFR depleted electrons from the inter-system chain, so that the subsequent saturating pulse oxidised P700 maximally (Siebke et al. 1997). While the sFR was on, at *T *= 9.0 s, SP (~ 10,000 µmol photons m^−2^ s^−1^) was applied for 200 ms by a pulse from Dual-PAM/F and a fourth terminal of the pulse/delay generator, yielding the maximally oxidised $P^{\prime}_{\text{m}}$ signal (where $P^{\prime}_{\text{m}}$ is the maximum P700^+^ signal in AL) in Fig. S 2B. Finally, AL was turned off by the electronic shutter (at *T *= 9.016 s). Data acquisition continued for 1200 ms after cessation of AL to obtain the baseline corresponding to complete re-reduction of P700^+^. Y(I) was then calculated by the Dual-PAM from the complimentary PSI quantum yields of non-photochemical energy dissipation Y(ND) and Y(NA):

1 $${\text{Y}}\left( {\text{I}} \right) = 1 - {\text{Y}}\left( {\text{ND}} \right) - {\text{Y}}\left( {\text{NA}} \right)$$

Y(ND) and Y(NA) were directly determined by the saturation pulse method. Y(ND) represents the fraction of overall P700 that is oxidised in a given state. It is calculated as:

2 $${\text{Y}}\left( {\text{ND}} \right) = 1 - {\text{P}}700_{{{\text{red}} .}}$$

where P700_red_. is the fraction of P700 in the reduced state. As determination of P700_red_. by the saturation pulse method requires previous *P*_m_ determination, the same also holds for Y(ND) determination. Y(NA), on the other hand, represents the fraction of overall P700 that cannot be oxidised by a saturation pulse in a given state due to lack of available acceptors. It is calculated as:

3 $${\text{Y}}\left( {\text{NA}} \right) = \frac{{\left( {P_{\text{m}} - P^{\prime}_{\text{m}} } \right)}}{{P_{\text{m}} }}$$

ETR1 was then calculated as:

4 $${\text{ETR}}1 = {\text{Y}}\left( {\text{I}} \right) \times I \times 0.85 \times f_{\text{I}}$$

where *I* is the irradiance, 0.85 is the assumed absorptance and $f_{\text{I}}$ is the assumed fraction of absorbed white light partitioned to PSI.

### Determination of $f_{\text{I}}$ and calculation of CEF

Two techniques were compared in this study to determine $f_{\text{I}}$: (1) the use of CEF inhibitors and (2) simultaneous measurement of linear electron flux $\left( {{\text{LEF}}_{{{\text{O}}_{ 2} }} } \right)$ by Chl fluorescence and gross oxygen evolution rate under white actinic light of very low irradiances. In the first technique, CEF is assumed to be zero; thus, ETR2 is supposed to be approximately equal to ETR1. In the second technique, linear electron fluxes measured by Chl fluorescence and oxygen evolution are assumed to be equal.

To obtain $f_{\text{I}}$ using the first technique, $f_{\text{I}}$ was determined by inhibiting CEF with the use of antimycin A and TTFA. The cut end of the leaf was dipped into 200 µM antimycin A/200 µM TTFA/H_2_O solution (with a trace of ethanol) and allowed to absorb the solution in darkness overnight before measurement. Inhibitor concentration taken up by the leaf was calculated as:

5 $${\text{Inhibitor concentration}} = \frac{{\left( {{\text{Volume of consumed solution}} 200 \mu {\text{M}}} \right) }}{{\left( {{\text{Leaf fresh weight}} \times 0.9} \right)}}$$

where 0.9 represents the 90% water content of the leaf. Discs were collected and used for Y(I)-based measurement of ETR1 after absorbing ≥ 200 µM of each inhibitor. Assuming that:

6 $${\text{ETR}}1 = {\text{LEF}}_{{{\text{O}}_{ 2} }}$$

then

7 $${\text{LEF}}_{{{\text{O}}_{ 2} }} = {\text{Y}}\left( {\text{I}} \right) \times I \times 0.85 \times f_{I}$$

$f_{I}$ was then calculated as:

8 $$f_{\text{I}} = \frac{{{\text{LEF}}_{{{\text{O}}_{ 2} }} }}{{{\text{Y}}\left( {\text{I}} \right) \times I \times 0.85}}$$

CEF in leaf in the absence of inhibitors was then calculated as:

9 $${\text{CEF}} = {\text{ETR}}1 - {\text{LEF}}_{{{\text{O}}_{ 2} }}$$

To obtain $f_{\text{I}}$ using the second technique, the fraction of absorbed white light partitioned to PSII, $f_{\text{II}} ,$ was first estimated in leaf discs of different species by measuring the photochemical yield of PS II, Y(II), by Chl fluorescence and the gross oxygen evolution rate simultaneously at low irradiance (50, 100, 200, 300 µmol photons m^−2^ s^−1^) and high *p*CO_2_ (4%). Chl fluorescence was measured with the fluorescence single channel in SP-analysis mode of the Dual-PAM software when the gas exchange signals were all stable. The steady-state fluorescence yield (*F*_s_) was first monitored continuously under low irradiances, and a 300-ms pulse of saturating light (~ 10,000 µmol photons m^−2^ s^−1^) was supplied to determine maximum variable fluorescence $\left( {F^{\prime}_{\text{m}} } \right)$. Y(II) at the steady state was defined as $\left( {F^{\prime}_{\text{m}} {-}F_{\text{s}} } \right)/F^{\prime}_{\text{m}}$, as proposed by Genty et al. (1989). ETR2 was then calculated as:

10 $${\text{ETR}}2 = {\text{Y}}\left( {\text{II}} \right) \times I \times 0.85 \times f_{\text{II}}$$

Under low actinic irradiance (< 500 µmol photons m^−2^ s^−1^), Kou et al. (2013a) showed that ETR2 can be equated to ${\text{LEF}}_{{{\text{O}}_{ 2} }}$; further, at low actinic irradiance, the matching of ETR2 with ${\text{LEF}}_{{{\text{O}}_{ 2} }}$ is independent of the spectral distribution of the excitation light (Zhang et al. 2018). ${\text{LEF}}_{{{\text{O}}_{ 2} }}$ in the present study is the gross oxygen evolution rate during illumination recorded by MIMS multiplied by four (since four electrons are released for each oxygen molecule evolved). Assuming that:

11 $${\text{ETR}}2 = {\text{LEF}}_{{{\text{O}}_{ 2} }}$$

then

12 $${\text{LEF}}_{{{\text{O}}_{ 2} }} = {\text{Y}}\left( {\text{II}} \right) \times I \times 0.85 \times f_{\text{II}}$$

allowing *f*_II_ to be evaluated.$f_{\text{I}}$ was then calculated as:

13 $$f_{\text{I}} = 1 - f_{\text{II}}$$

The ETR2 obtained by the gross oxygen evolution rate is based on whole-tissue measurement and can be validly compared with ETR1 obtained from Y(I). This is because the P700^+^ signal is also a whole-tissue measurement, by virtue of the fact that the measuring beams at 820 and 870 nm are only weakly absorbed by the leaf tissue and are, therefore, multiply scattered in the tissue until they are finally absorbed; subtraction of ${\text{LEF}}_{{{\text{O}}_{ 2} }}$ from ETR1 is then valid, as both refer to the same leaf tissue (Fan et al. 2016).

### Data analysis

For each variable, four replicates (independent samples) were obtained for the two light treatments. The results were subjected to analysis of variance, and the means were compared by the Tukey test at 5% probability.

## Results

### Comparing two methods for estimating the fraction of absorbed light partitioned to PSI (*f*_I_)

Estimation of CEF from ETR1 requires prior information on *f*_I_, which, in turn, requires a situation where CEF is small or negligible. This can be achieved by: (1) using inhibitors of CEF such as antimycin A and TTFA or (2) using low irradiance to drive LEF to produce minimal CEF. The efficiency of these two methods was evaluated by concurrently measuring fluorescence and P700 signals with mass spectrometric measurements of gross O_2_ evolution in a closed leaf chamber (Figs. S 1 and S 2).

Using the first method, leaf discs of C_4_ grasses *P. miliaceum* and *M. maximus* were infiltrated with ~ 200 µM solution of each inhibitor and Y(I) was measured using Dual-PAM/F under increasing irradiance from 100 to 2000 µmol photons m^−2^ s^−1^. Assuming that CEF was completely inhibited, ETR1 would approximately equal ${\text{LEF}}_{{{\text{O}}_{ 2} }}$ as in Eq. (6). Then, *f*_I_ can be estimated by equating ETR1 with ${\text{LEF}}_{{{\text{O}}_{ 2} }}$ and using measured Y(I) as in Eqs. (7) and (8). If CEF was inhibited in this method, *f*_I_ would be independent of irradiance (Kou et al. 2013a, b). However, *f*_I_ decreased with increasing irradiance in all inhibitor-treated leaf discs (Table S 1). In addition, ${\text{LEF}}_{{{\text{O}}_{ 2} }}$ of treated leaf discs was lower compared to untreated discs, probably due to the unknown, non-specific effect of inhibitors in photosynthetic electron transport. This could affect ETR1 calculation and underestimate *f*_I_ due to the side effects of high concentration of CEF inhibitors on photosynthesis. Consequently, we considered that this method was unreliable for *f*_I_ estimation.

**Fig. 1:**
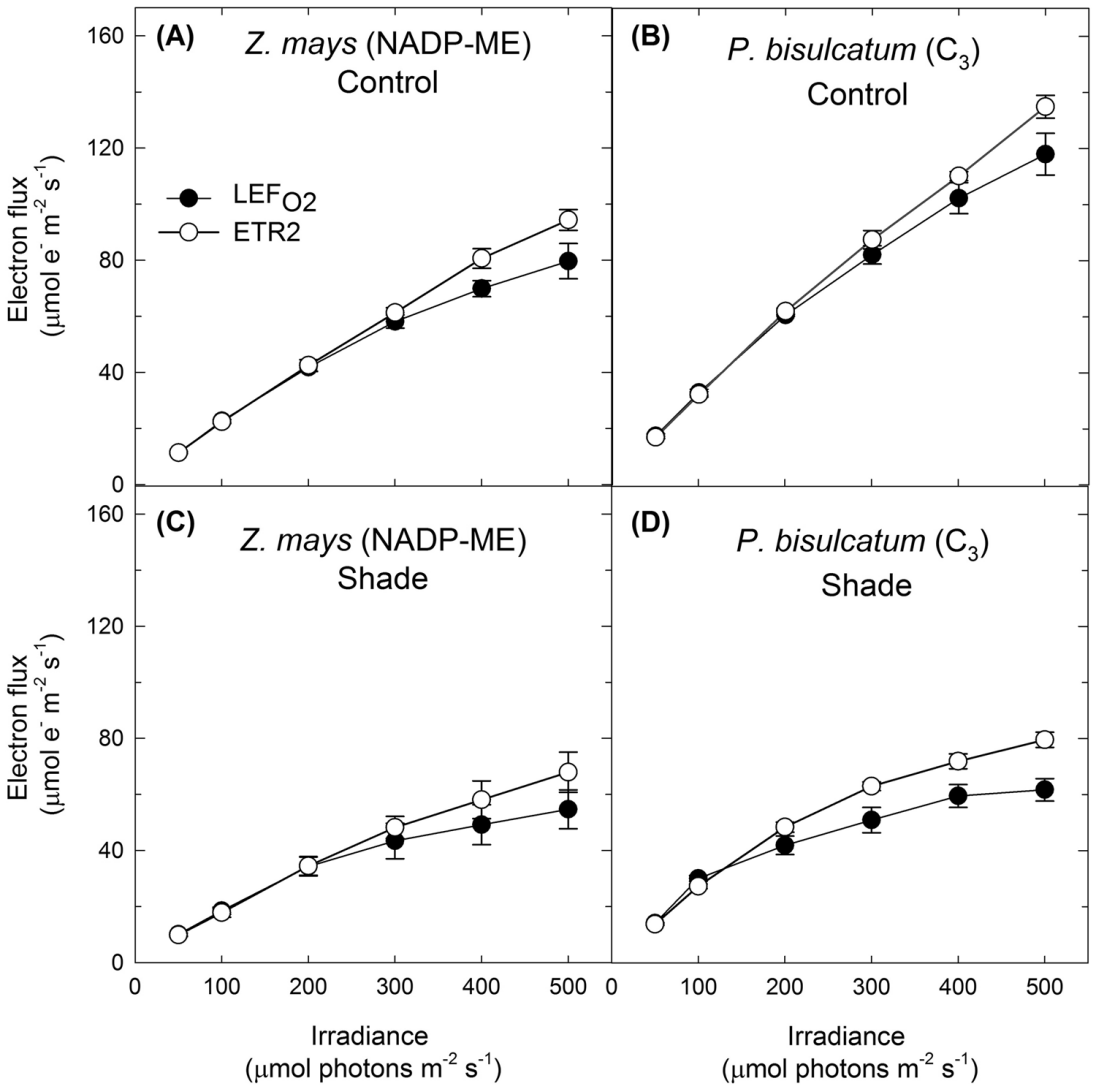
Electron fluxes through PSII in response to measurement irradiance calculated in two different ways in leaf discs of control (**a**) and shade-grown (**c**) *Zea mays* (NADP-ME) and control (**b**) and shade-grown (**d**) *Panicum bisulcatum* (C_3_ grass). ${\text{LEF}}_{{{\text{O}}_{ 2} }}$ (the gross oxygen evolution rate multiplied by four) represents the linear electron flux through both photosystems; ETR2 is the measure of electron flux through PSII based on Chl fluorescence emitted from a certain depth in leaf tissue calculated using experimentally derived *f*_II_. Measurements were taken under the temperature of 28 °C and high *p*CO_2_ condition (4%). Values are mean ± S.E. (*n* = 4 leaf discs)

The second method used Y(II) obtained by simultaneously measuring chl fluorescence and gross O_2_ evolution rate at low irradiance and high *p*CO_2_. This method is considered to be non-destructive in comparison with the use of CEF inhibitors (Table S 1). Since MIMS was directly connected to a Dual-PAM, simultaneous measurements of O_2_ evolution and the quantum yield of the photochemical reaction at PSII or PSI were possible which made us easily calculate light partitioning and various electron fluxes in leaf (Fig. 1a–d). The method is also mechanistic and quantitative which assumes a linear relationship between the gas exchange and photochemical yields which was adopted in a number of studies (Beckmann et al. 2009; Fan et al. 2016; Kono et al. 2014; Kou et al. 2013b, 2015; Laisk et al. 2014; Laisk and Loreto 1996; Loreto et al. 2009; Miyake and Yokota 2000; Miyake et al. 2004, 2005). At ≤ 300–500 µmol photons m^−2^ s^−1^, CEF was assumed to be negligible in CO_2_-enriched air. Under these conditions, Kou et al. (2013a) showed that ETR2 roughly matched ${\text{LEF}}_{{{\text{O}}_{ 2} }}$ in spinach. We equated ${\text{LEF}}_{{{\text{O}}_{ 2} }}$ to ETR2 as in Eq. (11). Then, using Y(II) obtained from Dual-PAM/F, *f*_II_ was obtained according to Eq. (12) and subtracted from 1 to give *f*_I_. The estimated *f*_II_ derived from Y(II) measurement was used to calculate a new ETR2 and plotted against irradiance, as was ${\text{LEF}}_{{{\text{O}}_{ 2} }}$. Results showed that ETR2 of control and shade-grown *Z. mays* (NADP-ME) (Fig. 1a and c) and *P. bisulcatum* (C_3_ grass) (Fig. 1b and d) roughly matched ${\text{LEF}}_{{{\text{O}}_{ 2} }}$ at irradiance < 300–500 µmol photons m^−2^ s^−1^. Hence, this method was considered more reliable compared to the first at low irradiance and was subsequently used for *f*_I_ estimation of other species.

Utilising our second more reliable method, we calculated *f*_*I*_ for the species used in this study (Table 1). All control C_4_ species had estimated *f*_I_ of 0.6 except for PCK grass (*M. maximus*) which had 0.5. On the other hand, *f*_I_ of shade-grown C_4_ species remained constant except for NADP-ME grass (*P. antidotale*) which had 0.7 (Table 1). Control C_3_ grass (*P. bisulcatum*) had *f*_I_ of 0.4 which is lower compared to spinach (C_3_ model species), having *f*_I_ of 0.5 (Table 1). However, shade-grown C_3_ grass had *f*_I_ of 0.5. Other species such as liverwort (*M. polymorpha*) and fern (*Polypodium* sp.) had *f*_I_ of 0.5, while the two species of gymnosperms had lower *f*_I_ which was 0.4 for *G. biloba* and 0.3 for *W. nobilis* (Table 1). Overall, there was a significant species × treatment effect on both *f*_I_ and *f*_II_ (Table 2).

**Table 1:** An estimation of the fraction of absorbed light partitioned to PSI (*f*_I_) obtained by Chl fluorescence method measured under low irradiances, high *p*CO_2_ conditions (4%) and temperature of 28 °C in leaf of control and shade-grown C_3_, C_4_, gymnosperm, fern and liverwort species

Irradiance (µmol photons m^−2^ s^−1^)	*f*_I_ control	*f*_I_ shade
*Panicum bisulcatum* (*n* = 4) (C_3_)		
50	0.40 ± 0.02	0.51 ± 0.01
100	0.40 ± 0.01	0.47 ± 0.02
200	0.43 ± 0.01	0.59 ± 0.02
300	0.45 ± 0.02	0.61 ± 0.03
*Panicum miliaceum* (*n* = 4) (NAD-ME)		
50	0.60 ± 0.01	0.58 ± 0.02
100	0.58 ± 0.01	0.59 ± 0.01
200	0.62 ± 0.02	0.64 ± 0.02
300	0.67 ± 0.03	0.69 ± 0.02
*Megathyrsus maximus* (*n* = 4) (PCK)		
50	0.43 ± 0.01	0.54 ± 0.03
100	0.42 ± 0.00	0.56 ± 0.02
200	0.47 ± 0.01	0.63 ± 0.03
300	0.50 ± 0.01	0.68 ± 0.03
*Panicum antidotale* (*n* = 4) (NADP-ME)		
50	0.60 ± 0.02	0.62 ± 0.02
100	0.57 ± 0.02	0.65 ± 0.01
200	0.57 ± 0.02	0.68 ± 0.02
300	0.62 ± 0.01	0.73 ± 0.02
*Zea mays* (*n* = 8) (NADP-ME)		
50	0.58 ± 0.02	0.60 ± 0.01
100	0.57 ± 0.02	0.59 ± 0.02
200	0.58 ± 0.02	0.61 ± 0.03
300	0.60 ± 0.02	0.65 ± 0.03
Spinach (*n* = 5) (C_3_)		
50	0.49 ± 0.01	
100	0.51 ± 0.00	
200	0.50 ± 0.00	
300	0.50 ± 0.00	
*Ginkgo biloba* (*n* = 4) (gymnosperm)		
50	0.37 ± 0.01	
100	0.38 ± 0.01	
200	0.43 ± 0.00	
300	0.46 ± 0.01	
*Wollemi nobilis* (*n* = 4) (gymnosperm)		
50	0.22 ± 0.03	
100	0.31 ± 0.04	
200	0.49 ± 0.07	
300	0.49 ± 0.08	
*Polypodium* sp. (*n* = 4) (fern)		
50	0.48 ± 0.01	
100	0.54 ± 0.01	
200	0.61 ± 0.02	
300	0.59 ± 0.01	
*Marchantia polymorpha* (*n* = 4) (liverwort)		
50	0.44 ± 0.01	
100	0.49 ± 0.02	
200	0.57 ± 0.03	
300	0.61 ± 0.04	

Values are mean ± S.E.

**Table 2:** Summary of statistical analysis using two-way ANOVA for the effects of shade and species on various parameters collected for nine plants grown under natural light (~ 800 µmol photons m^−2^ s^−1^) and shaded (~ 300 µmol photons m^−2^ s^−1^) conditions

Parameter	Main effects (P)		Interactions (P)
	Species	Treatment	Species × treatment
Y(II)	0.021	0.000	0.014
Y(I) at low light	0.001	0.080	0.240
Y(I) at medium light	0.023	0.216	0.180
Y(I) at saturating light	0.084	0.704	0.184
*f*_I_	0.000	0.000	0.003
*f*_II_	0.000	0.000	0.003
${\text{LEF}}_{{{\text{O}}_{ 2} }}$ at low light (µmol m^−2^ s^−1^)	0.000	0.000	0.098
${\text{LEF}}_{{{\text{O}}_{ 2} }}$ at medium light (µmol m^−2^ s^−1^)	0.000	0.000	0.014
${\text{LEF}}_{{{\text{O}}_{ 2} }}$ at saturating light (µmol m^−2^ s^−1^)	0.000	0.000	0.140
ETR1 at low light (µmol m^−2^ s^−1^)	0.000	0.002	0.011
ETR1 at medium light (µmol m^−2^ s^−1^)	0.000	0.779	0.039
ETR1 at saturating light (µmol m^−2^ s^−1^)	0.000	0.210	0.122
CEF at low light (µmol m^−2^ s^−1^)	0.000	0.000	0.000
CEF at medium light (µmol m^−2^ s^−1^)	0.000	0.000	0.266
CEF at saturating light (µmol m^−2^ s^−1^)	0.000	0.000	0.341
CEF/${\text{LEF}}_{{{\text{O}}_{ 2} }}$ at low light	0.000	0.000	0.544
CEF/${\text{LEF}}_{{{\text{O}}_{ 2} }}$ at medium light	0.000	0.000	0.052
CEF/${\text{LEF}}_{{{\text{O}}_{ 2} }}$ at saturating light	0.001	0.000	0.294

Measurements were taken at low light (200 µmol photons m^−2^ s^−1^), medium light (1000 µmol photons m^−2^ s^−1^) and saturating light (2000 µmol photons m^−2^ s^−1^) under the temperature of 28 °C and high *p*CO_2_ condition (4%)

### The response of ETR1 and CEF in C_3_ and C_4_ grass species to shade

**Fig. 2:**
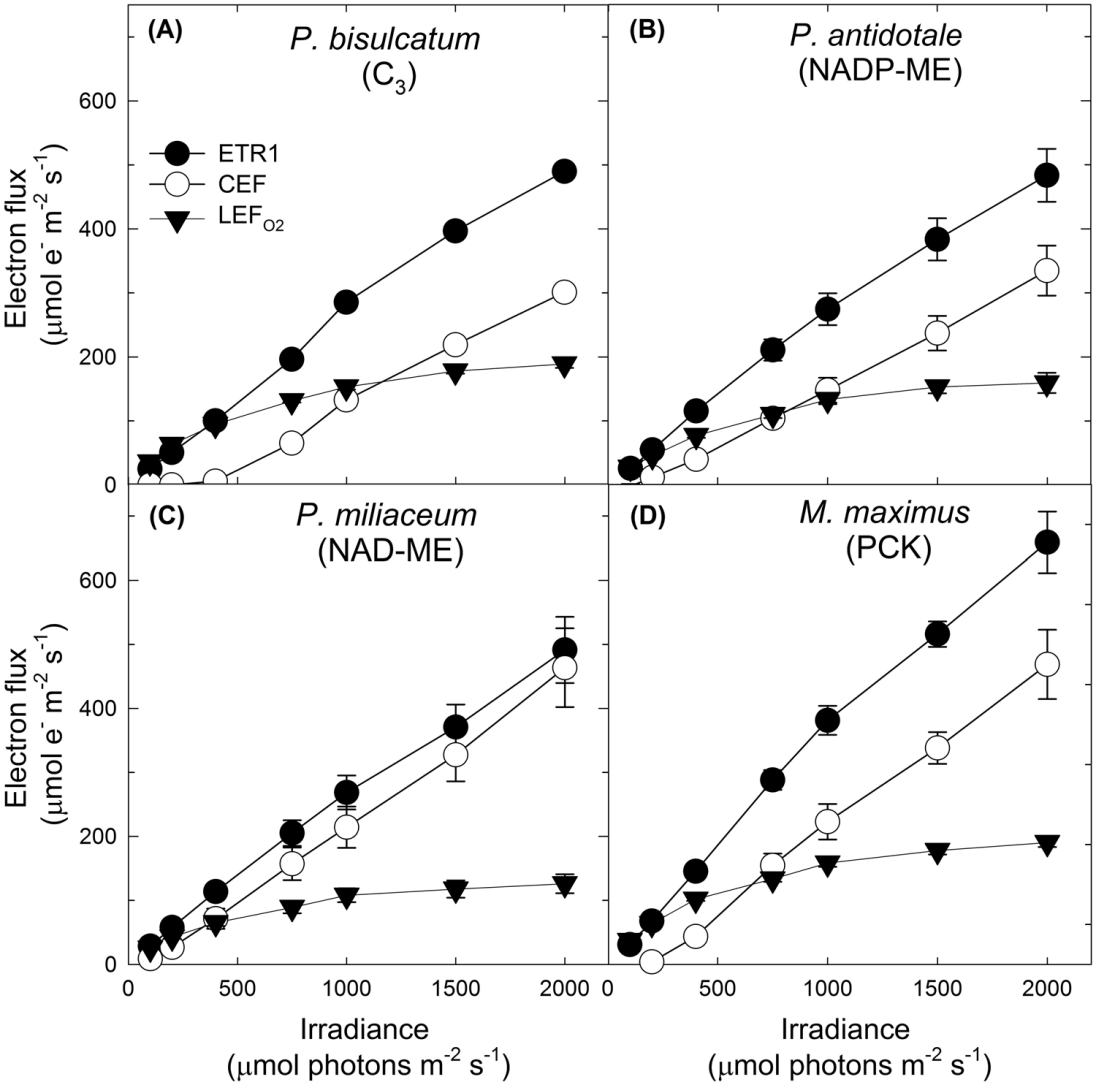
Various electron fluxes in response to irradiance in leaf discs of control **a***Panicum bisulcatum* (C_3_ grass); **b***Panicum antidotale* (NADP-ME grass); **c***Panicum miliaceum* (NAD-ME grass); and **d***Megathyrsus maximus* (PCK grass). ${\text{LEF}}_{{{\text{O}}_{ 2} }}$ (the gross oxygen evolution rate multiplied by four) represents the linear electron flux through both photosystems. ETR1 is the measure of electron flux through PSI calculated using experimentally derived *f*_I_. CEF represents the cyclic electron flux around PSI calculated by subtracting ${\text{LEF}}_{{{\text{O}}_{ 2} }}$ from ETR1. Measurements were taken under the temperature of 28 °C and high *p*CO_2_ condition (4%). Values are mean ± S.E. (*n* = 4 leaf discs)

**Fig. 3:**
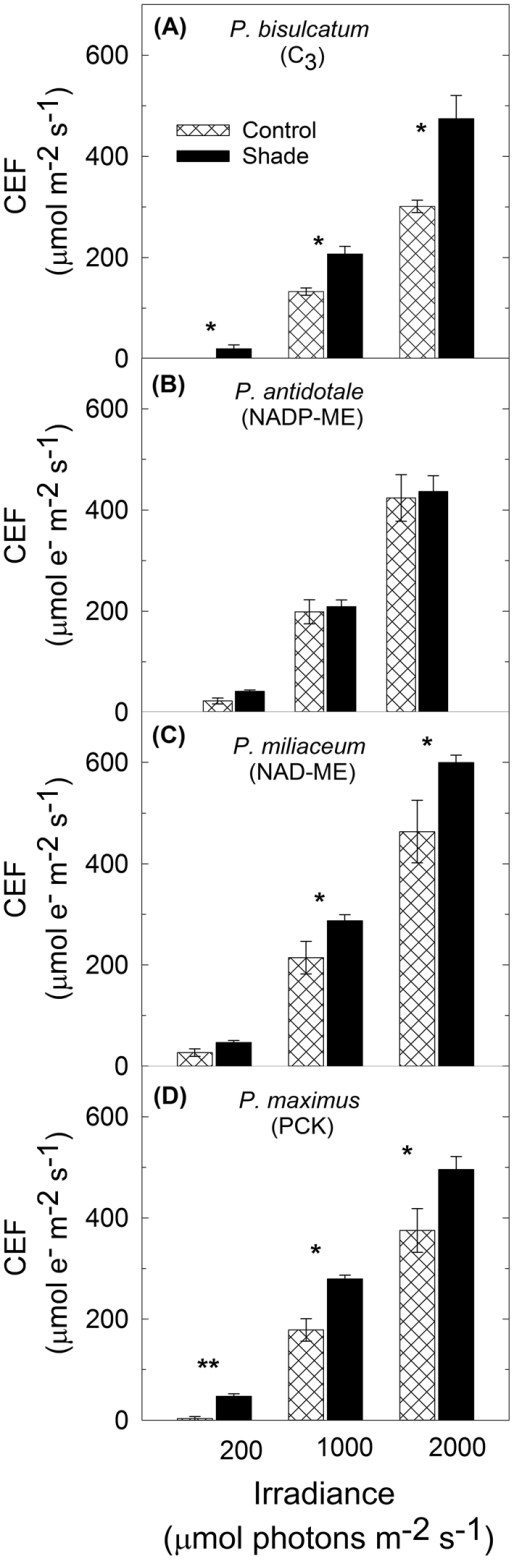
Cyclic electron flux around PSI (CEF) in response to low, medium and saturating irradiance (200, 1000, 2000 µmol photons m^−2^ s^−1^) measured under the temperature of 28 °C and high *p*CO_2_ condition (4%) in leaf discs of control and shade-grown **a***Panicum bisulcatum* (C_3_ grass); **b***Panicum antidotale* (NADP-ME grass); **c***Panicum miliaceum* (NAD-ME grass); and **d***Megathyrsus maximus* (PCK grass). Each column represents the mean ± S.E. of species (*n* = 4 leaf discs) at each light intensity. Statistical significance levels (*t* test) for the growth condition within each species and measurement light intensity are shown, and they are: *p < 0.05; **p < 0.01; ***p < 0.001

Using our estimated *f*_I_ values to calculate ETR1, it was observed that ETR1 was higher in control NADP-ME (Fig. 2b) and NAD-ME (Fig. 2c) grasses by ~ 20% and lower in control PCK (~ 9%) (Fig. 2d) and C_3_ (~ 16%) (Fig. 2a) grasses when compared to uncorrected ETR1 (calculated using *f*_I_ = 0.5). Using corrected ETR1, CEF was then calculated using Eq. (9). At ≥ 500 µmol photons m^−2^ s^−1^, CEF increased by ~ 32% and 38% in control NADP-ME (Fig. 2b) and NAD-ME (Fig. 2c) grasses and decreased by ~ 14% and ~ 28% in control PCK (Fig. 2d) and C_3_ (Fig. 2a) grasses, respectively. Overall, there was no significant species x treatment effect on ETR1, CEF and ${\text{LEF}}_{{{\text{O}}_{ 2} }}$ measured at saturating irradiance (2000 µmol photons m^−2^ s^−1^) (Table 2). However, when measured at low, medium and saturating irradiance (200, 1000 and 2000 µmol photons m^−2^ s^−1^), leaf discs of all shade-grown grass species had significantly higher CEF rates compared to the control counterparts (Fig. 3a–d), except for the NADP-ME grass which had no significant difference to the control (Fig. 3b).

When measured at low irradiance, CEF rates increased the most in shade-grown C_3_ grass (+ 1900%) (Fig. 3a) followed by PCK grass (+ 1395%) (Fig. 3D), NADP-ME grass (+ 86%) (Fig. 3b) and NAD-ME grass (+ 75%) (Fig. 3c) relative to the control counterparts. Highest increase in CEF rate measured at saturating irradiance was again observed in shade-grown C_3_ grass (+ 58%) (Fig. 3a) followed by PCK grass (+ 32%) (Fig. 3d), NAD-ME grass (+ 29%) (Fig. 3c) and NADP-ME grass (+ 3%) (Fig. 3b) species relative to the control counterparts.

Among shade-grown plants, CEF rates measured at medium irradiance (1000 µmol photons m^−2^ s^−1^) were ~ 35% higher in *P. miliaceum* (NAD-ME), *M. maximus* (PCK) and *Z. mays* (NADP-ME) relative to *P. bisulcatum* (C_3_) and *P. antidotale* (NADP-ME) (Tables 2 and S 2).

It was also observed that ${\text{LEF}}_{{{\text{O}}_{ 2} }}$ was significantly lower in all shade-grown species relative to the control counterparts when measured at medium irradiance (Tables 2 and S 2). However, ETR1 was not affected by the shade treatment in any of the species except for *P. antidotale*, where ETR1 decreased by 28% under shade (Tables 2 and S 2).

### Rates of CEF of other species in response to irradiance

**Fig. 4:**
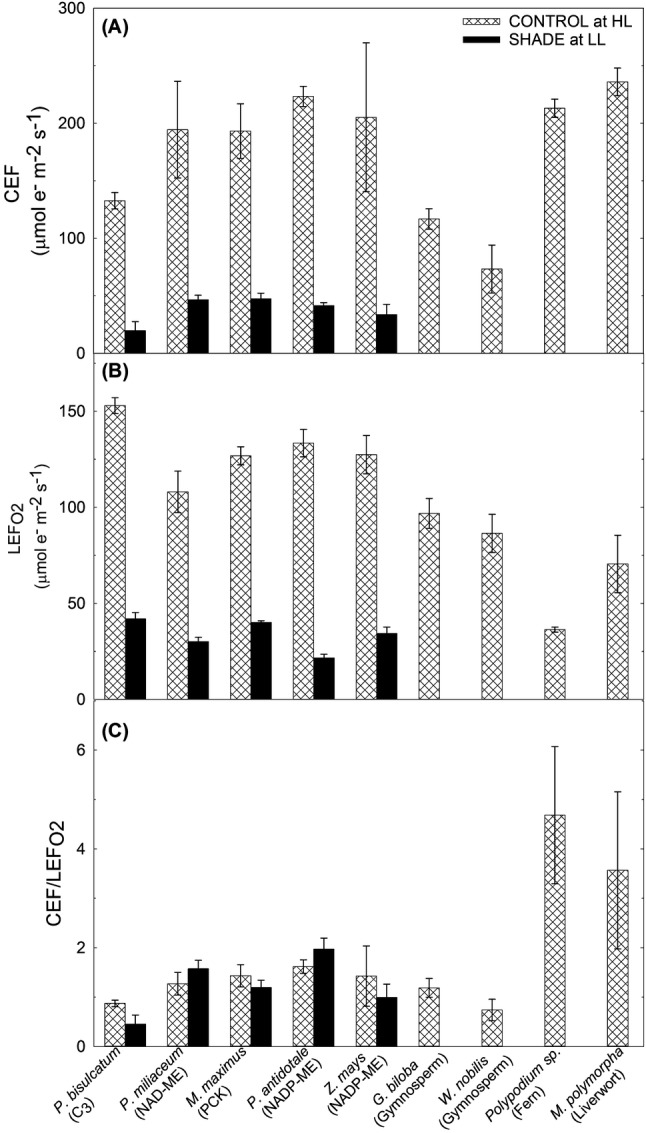
**a** Cyclic electron flux through PSI (CEF); **b** linear electron flux through both photosystems $\left( {{\text{LEF}}_{{{\text{O}}_{ 2} }} } \right)$; and **c** the ratio of CEF to ${\text{LEF}}_{{{\text{O}}_{ 2} }}$ of control plants measured at 1000 µmol photons m^−2^ s^−1^ (HL) and shade-grown plants measured at 200 µmol photons m^−2^ s^−1^ (LL). Measurements were taken under the temperature of 28 °C and high *p*CO_2_ condition (4%) in leaf discs of *Panicum bisulcatum* (C_3_ grass), *Panicum antidotale* (NADP-ME grass), *Panicum miliaceum* (NAD-ME grass), *Megathyrsus maximus* (PCK grass), *Zea mays* (NADP-ME), *Ginkgo biloba* (Gymnosperm), *Wollemi nobilis* (Gymnosperm), *Polypodium* sp. (Fern) and *Marchantia polymorpha* (Liverwort). Each column represents the mean ± S.E. of each species (*n* = 4 leaf discs)

${\text{LEF}}_{{{\text{O}}_{ 2} }}$, ETR1 and CEF rates of all control species increased approximately linearly with irradiance (Tables 2 and S 2). Operation of CEF at low irradiance (200 µmol photons m^−2^ s^−1^) was almost negligible in control C_3_ grass and gymnosperm species (Tables 2 and S 2). This is because of the rate of ${\text{LEF}}_{{{\text{O}}_{ 2} }}$ almost equalled ETR1 (Table S 2), suggesting that all electrons from PSII were transferred to acceptors in PSI in these species without cycling around PSI. CEF started to operate between 400 and 750 µmol photons m^−2^ s^−1^ in C_3_ grass and gymnosperm species, while operation of CEF in other species started at much lower irradiances (Table S 2). Among all control C_4_ species, rapid stimulation of CEF under low irradiance (< 400 µmol photons m^−2^ s^−1^) was observed in NADP-ME and NAD-ME species, while CEF of PCK grass was stimulated at much higher irradiance (Table S 2). Among all species grown under high light, all grass species including *Z. mays* had higher ${\text{LEF}}_{{{\text{O}}_{ 2} }}$ compared to the gymnosperms, liverwort and fern when measured at medium irradiance (Fig. 4b; Tables 2 and S 2). NADP-ME and NAD-ME grasses had the highest rates of CEF, which was not significantly different among the other species (Fig. 4b; Tables 2 and S 2). All shade-grown plants measured at low irradiance had lower rates of CEF and ${\text{LEF}}_{{{\text{O}}_{ 2} }}$ compared to the control counterparts measured at medium irradiance (Fig. 4a, b; Tables 2 and S 2). This suggests that electron fluxes of shade-grown plants operate at a slower rate under their growing light conditions in comparison with control plants. The ratio of CEF to ${\text{LEF}}_{{{\text{O}}_{ 2} }}$ was generally higher in C_4_ relative to C_3_ grass under both control and shade conditions (Fig. 4c). In addition, the CEF to ${\text{LEF}}_{{{\text{O}}_{ 2} }}$ ratio was not significantly different among the C_4_ grasses under either control or shade conditions (Fig. 4c). Liverwort and fern species had higher CEF to ${\text{LEF}}_{{{\text{O}}_{ 2} }}$ ratio relative to all other species under grown under high light (Fig. 4c). Overall, no significant species × treatment effect on CEF to ${\text{LEF}}_{{{\text{O}}_{ 2} }}$ ratio under low, medium and saturating light (Table 2).

## Discussion

In this study, we tested two methods to calculate the light partitioning between PSI and PSII (*f*_I_ and *f*_II_, respectively) in several plant species by combining P700 measurement using a Dual-PAM and LEF measurement using MIMS. Given that our species of interest are not widely studied, we included spinach (C_3_ model species) and maize (C_4_ model species) in our study to compare our values with the literature. One method was more reliable and we adopted it to determine CEF around PSI.

### The use of CEF inhibitors is unreliable for f_I_ estimation in leaves of C_4_ grasses

The concentration of the CEF inhibitors that should infiltrate the leaf must be ≥ 200 µM. At this concentration, Kou et al. (2013a) observed that antimycin A had no effect on ${\text{LEF}}_{{{\text{O}}_{ 2} }}$ assayed by O_2_ evolution and largely abolished CEF in spinach leaf discs. However, the same was not observed in some C_4_ grass leaves examined. After allowing the leaf to take up the inhibitor solution overnight, the leaf started to dry out and ${\text{LEF}}_{{{\text{O}}_{ 2} }}$ values were lower compared to the untreated leaf (Table S 1), possibly due to the unknown, non-specific effect of the inhibitors in many mechanisms of light reactions in the chloroplasts. The combination of these two potent CEF inhibitors might have multiple effects on photosynthesis. An example is from the study of Horton et al. (1991) where they found that antimycin A prevents LHCII aggregation which inhibits the process of excess excitation energy dissipation as heat (qE). It was also observed that TTFA can inhibit photosynthetic electron transport in and around PSII complex in spinach as measured from chlorophyll fluorescence parameters (Ikezawa et al. 2002).

Aside from these reasons, it was also decided not to measure *f*_I_ under high irradiance for C_4_ plants because of the amount of charge recombination occurring in both mesophyll and bundle sheath chloroplasts (Takahashi et al. 2013; Kou et al. 2015). This direct charge recombination can keep P700 more reduced even CEF was inhibited. In this case, Y(I) would be greater than in the absence of direct charge recombination and smaller *f*_I_ values under increasing irradiance (Table S 1). This phenomenon was also observed in low-light-grown *Arabidopsis* that lacks NDH which still exhibited a substantial ∆Flux at high irradiance even in the presence of antimycin A, attributable to charge recombination in PSI and/or the Mehler reaction (Kou et al. 2015). Another possibility for P700 reduction is the reduction of stromal donors by ascorbate and malate under aerobic conditions with weak far-red light. It was shown by Ivanov et al. (2005) that addition of these metabolites strongly stimulated the development of a proton gradient in thylakoids of maize under aerobic conditions in the absence of DCMU, suggesting the physiological role in the activation of CEF around PSI.

### Comparison of f_I_ across a wide range of species and in response to shade

Higher value of *f*_I_ in C_4_ species compared to C_3_ species (Tables 1 and 2) validated the hypothesis that more excitation energy is distributed to PSI compared to PSII in C_4_ species. This was expected because leaves of C_4_ plants contain two types of photosynthetic cells, mesophyll and bundle sheath cells, which are quite distinctly organised, both structurally and functionally having varying PSI/PSII ratio depending on subtypes (Ghannoum et al. 2005; Romanowska and Albertsson 1994; Romanowska et al. 2008; Romanowska and Drożak 2006; Drozak and Romanowska 2006). For the representative species of gymnosperm, liverwort and fern, the higher values of *f*_II_ compared to *f*_I_ suggest the greater amount of PSII components relative to PSI in mesophyll chloroplasts. These shifted *f*_I_ and *f*_II_ values might be related to processes involving flavodiiron proteins (Flv) and other PSI protection mechanisms in these species. As such, they may utilise greater pseudo-cyclic pathways to balance energy requirements and inputs and alleviate photo-oxidative damage (Allahverdiyeva et al. 2015; Hanawa et al. 2017; Ilík et al. 2017; Noridomi et al. 2017; Shimakawa et al. 2017; Shirao et al. 2013). However, further experiments involving morphological and biochemical examinations of the leaf should be done to quantify functional PSI and PSII contents as well as the antenna size of each photosystem in these species.

Growth irradiance is also believed to affect the distribution of excitation energy by modulating the composition of light-harvesting antennas of PSI and PSII (Anderson 1986; Huner et al. 2003; Tanaka and Melis 1997). Growth under low light promotes large PSI and PSII antenna size, whereas growth under high light generates a small photosynthetic unit (Akoumianaki-Ioannidou et al. 2004; Huner et al. 2003; Leong and Anderson 1984). In C_3_ plants, the value of *f*_II_ was expected to be greater than that of *f*_I_ because PSII absorbs more light than PSI and this proportion increases with adaptation to shade based on the study of Evans (1986). But the result of this study showed that in the C_3_ model species spinach, almost 50% of the absorbed light was partitioned to PSII and the other 50% to PSI (Table 1) which is consistent to the study of Fan et al. (2016) and Kou et al. (2013a), thus validating the reliability of this method. This partitioning is different in the leaf of control C_3_ grass (*P. bisulcatum*) examined where almost 60% of light was partitioned to PSII and this partitioning decreased when grown under shade. In the case of C_4_ plants, a large fraction of the absorbed light energy (~ 60%) was partitioned to PSI in the leaf of control plants and slightly increased in shade-grown plants. Several studies showed that adaptation to shade can increase *f*_II_ because of the lowering of the chlorophyll *a/b* ratio which will increase the amount of chlorophyll associated with PSII relative to chlorophyll associated with PSI (Walters and Horton 1995; Hogewoning et al. 2012; Murakami et al. 2016, 2017; Chow et al. 1990). However, the results obtained here were different from their findings for both shade-grown C_3_ and C_4_ grasses. An increase in PSI content has previously been observed by Bailey et al. (2001), but this change occurred only under very low irradiance with light intensities below 100 μmol photons m^−2^ s^−1^. The slight decrease in *f*_II_ values of shade-grown plants (Table 1) can also be attributed to the light consumption brought about by the accessory pigment content of the photosynthetic complexes which were altered during shade acclimation (Laisk et al. 2014).

### CEF at increasing irradiance

The very low CEF rate observed at ≤ 300 µmol photons m^−2^ s^−1^ (Figs. 2a–d, 3a–d and Tables 2 and S 2) is because Calvin cycle was able to use the majority of NADPH at low irradiance, leaving little spare reduced ferredoxin for poising CEF. At maximum ${\text{LEF}}_{{{\text{O}}_{ 2} }}$, however, more reduced ferredoxin would be available for competition between NADP^+^ reduction and poising of CEF (Kou et al. 2013a; Okegawa et al. 2008) and CEF was larger than ${\text{LEF}}_{{{\text{O}}_{ 2} }}$ (Fig. 2a–d, Table S 2). This can be attributed to spectral distribution of the actinic light used in this study which favoured CEF over LEF. The actinic light from the halogen lamp used induced CEF above 500 µmol photons m^−2^ s^−1^ in control C_3_ grass and above 300 µmol photons m^−2^ s^−1^ in control C_4_ grasses. However, CEF was induced at lower irradiance (≤ 200 µmol photons m^−2^ s^−1^) in shade-grown C_3_ and C_4_ grasses (Fig. 3a–d; Table S 2), suggesting the formation of more reduced ferredoxin under low light and that the Calvin–Benson cycle started to get saturated with NADPH. This demonstrates the significant effect of the spectral distribution of actinic light on the CEF being investigated.

### Induction of CEF among different species (C_3_ and C_4_ grasses, gymnosperms, ferns and liverwort) under high light

Although no significant difference was observed between the CEF of control species measured at medium irradiance (Fig. 4c; Table S 2), CEF started to operate between 400 and 750 µmol photons m^−2^ s^−1^ in C_3_ grass and gymnosperm species while operation of CEF in other species started at much lower irradiance, suggesting the greater capacity for CEF in C_4_ and fern species (Table S 2). Since it is widely known that CEF is crucial for a proper balance of NADPH and ATP in the thylakoid stroma of photosynthetic organisms (Golding and Johnson 2003; Hatch 1987; Huang et al. 2012; Johnson 2011; Kramer and Evans 2011; Miyake 2010; Munekage et al. 2004; Rumeau et al. 2007; Shikanai 2007; Takabayashi et al. 2005; Takahashi et al. 2009), differences in the capacity for CEF can be due to differences in the energy requirement among species. In C_4_ plants, both C_3_ and C_4_ cycles are functional, thereby increasing the energetic cost of assimilating CO_2_ relative to that in C_3_ plants under varying irradiances.

Little is known about the energy requirements of ferns and liverworts and their capacities for CEF under varying irradiances. However, early onset of CEF under low irradiance in these species (Table S 2) suggests that it served as a mechanism to protect the photosynthetic apparatus from photodamage since CEF can generate a ∆pH across the thylakoid membrane through increased electron transfer from PSI back to plastoquinone, thus activating NPQ under intense radiation (Carlquist and Schneider 2001; Watkins et al. 2007). Induction of CEF in gymnosperms at higher irradiance in comparison with other species (Table S 2) might be due to the ecophysiological traits of these species. Gymnosperms commonly grow in the mid- to high-latitude regions of the Northern Hemisphere where severe climatic conditions such as chilling temperatures are often experienced. As a result, they may be required to be more flexible than angiosperms to control photosynthesis according to surrounding environmental conditions (Shirao et al. 2013).

### Capacity for CEF among the C_4_ subtypes

Much rapid stimulation of CEF at low irradiance (< 400 µmol photons m^−2^ s^−1^) in NADP-ME and NAD-ME species in comparison with PCK grass (Table S 2) can be due to differences in the energy requirements among subtypes. For example, in NADP-ME species, BSC require more ATP than MC. This assumption is supported by the findings that the BS chloroplasts of most NADP-ME species either completely lack or have less grana with little activity of PSII, which is indispensable for the production of ATP and NADPH in LEF (Chapman et al. 1980; Gutierrez et al. 1974; Hatch 1987; Kanai and Edwards 1999; Romanowska et al. 2008; Woo et al. 1970).

### Induction of CEF in shade-grown C_3_ and C_4_ species

The intensity of light under which plants grow has a significant effect on CEF (Miyake et al. 2005). Highest increase in CEF rate under low and high irradiances was observed in the shade-grown C_3_ grass (Fig. 3a, Table S 2), suggesting that C_3_ grass species is more efficient in maintaining a balance in the ATP/NADPH ratio under low-light conditions and can dissipate excess light energy harmlessly as heat under saturating light condition. This result also suggests that the induction of CEF in these shade-grown plants may serve as a photoprotective mechanism or to generate additional ATP switching from LEF to CEF as part of the acclimation strategy since it was shown that shade down-regulated ${\text{LEF}}_{{{\text{O}}_{ 2} }}$ in all species (Fig. 4b, Table S 2). It has been shown that shade-grown *Arabidopsis* developed high PSI/PSII ratio in leaves which is preferentially involved in CEF to generate ATP, suggesting that this may be a way in which cells make the best use of the light available under such conditions (Joliot and Joliot 2006). However, it was shown by Miyake et al. (2005) that tobacco plants exposed to high light have greater capacity for both CEF and NPQ when compared with plants grown under low light. They have suggested that the main role of CEF in plants acclimated to high light is to dissipate excess light energy through NPQ when illuminated at high irradiance. Under low light, the rate of photosynthesis of high light acclimated plants tends to be limited by the rate of ATP production rather than by the rate of NADPH production. Therefore, it was assumed that CEF assisted with ATP synthesis under weaker light in control plants (Yamori et al. 2011). By contrast, for plants acclimated to low light, the rates of photosynthesis and photorespiration are expected to be low. Consequently, they should have reduced demand for CEF-dependent ATP regeneration. Thus, Yamori et al. (2011) speculated that, in plants exposed to low levels of light, the relatively low CEF activity corresponds to the ATP demand by primary metabolisms. Their results indicate that CEF primarily assists in maintaining a balance in the ATP/NADPH ratio under sub-saturating light conditions but tends to mainly participate in photo-protection for PSI and PSII under saturating light conditions which can be true for the species of grasses used in our experiment.

No significant difference was observed in the capacity for CEF between control and shade-grown NADP-ME grass under low, medium and saturating irradiance (Fig. 3b, Tables 2 and S 2), suggesting that CEF-dependent generation of ΔpH mainly contributed to ATP synthesis under those levels of irradiance in control and shade-grown plants. This result somehow confirmed the findings of Sonawane et al. (2018) using several species of C_4_ grasses across subtypes. They showed that NAD-ME and to a lesser extent PCK species were generally outperformed by NADP-ME species. This response was underpinned by a more efficient CCM and quantum yield in NADP-ME.

## Conclusion

In this study, we developed a reliable method to calculate the light partitioning between PSI and PSII (*f*_I_ and *f*_II_, respectively) by combining P700 measurement using a Dual-PAM and LEF measurement using MIMS. We applied this method to estimate *f*_I_ for several plant species to determine whether *f*_I_ deviates from what is widely assumed (*f*_I_ = 0.5) in the literature. C_4_ grasses had *f*_I_ of 0.6 which is higher than what is usually assumed. C_3_ grass had *f*_I_ of 0.4 which is lower compared to the model C_3_ species. Other species such as liverwort and fern had *f*_I_ of 0.5, while gymnosperms had lower. However, it was also shown that these values can change depending on the growing conditions such as irradiance. Cyclic electron flow was negligible at very low irradiance; it was generally higher in C_4_ grasses and lower in gymnosperms. The values obtained here can be used to correctly quantify CEF and further used for photosynthesis modelling.

## Electronic supplementary material

Below is the link to the electronic supplementary material.

Supplementary material 1 (DOCX 300 kb)
