# Supplementary material for: Cyclic electron flow and light partitioning between the two photosystems in leaves of plants with different functional types
Source: Photosynth Res. 2019 Sep 13;142(3):321–34. doi: 10.1007/s11120-019-00666-1 (PMC6874625; doi:10.1007/s11120-019-00666-1)
Supplement: Supplementary file 1 — Supplementary material 1 (DOCX 300 kb) [file 11120_2019_666_MOESM1_ESM.docx]

Table S 1. An estimation of the fraction of absorbed light partitioned to PSI (*f*_I_) under increasing irradiances obtained by inhibiting CEF in leaf of control *Panicum miliaceum* (NAD-ME grass) and *Megathyrsus maximus* (PCK grass) by infiltration with 200 µM antimycin A: thenoyltrifluoroacetone (TTFA) solution. LEF_O2_ is the linear electron flux determined by the gross rate O_2_ evolution measured under the temperature of 28^o^C and high *p*CO_2_ condition (4%), Y(I) is the photochemical yield of PSI, Y(ND) is a measure of PSI donor side limitation and Y(NA) is a measure of PSI acceptor side limitation. Values are means ± s.e. (*n* = 4 leaves).

|  | Irradiance (µmol photons m^-2^ s^-1^) | Untreated | | | | |  | Treated | | | | |
| --- | --- | --- | --- | --- | --- | --- | --- | --- | --- | --- | --- | --- |
|  |  | LEF_O2_ (µmol e^-^ m^-2^ s^-1^) | Y(I) | Y(ND) | Y(NA) | *f*_I_ |  | LEF_O2_ (µmol e^-^ m^-2^ s^-1^) | Y(I) | Y(ND) | Y(NA) | *f*_I_ |
| *Panicum miliaceum* (NAD-ME) | 100 | 26.4 ± 1.3 | 0.68 ± 0.04 | 0.22 ± 0.02 | 0.10 ± 0.06 | 0.58 ± 0.01 |  | 24.2 ± 1.4 | 0.68 ± 0.02 | 0.20 ± 0.02 | 0.11 ± 0.03 | 0.42 ± 0.01 |
|  | 200 | 43.0 ± 1.6 | 0.67 ± 0.05 | 0.21 ± 0.02 | 0.06 ± 0.01 | 0.62 ± 0.02 |  | 34.7 ± 2.7 | 0.69 ± 0.02 | 0.22 ± 0.02 | 0.08 ± 0.02 | 0.30 ± 0.02 |
|  | 400 | 64.0 ± 1.8 | 0.65 ± 0.04 | 0.26 ± 0.02 | 0.09 ± 0.06 | 0.67 ± 0.03 |  | 49.4 ± 2.5 | 0.68 ± 0.02 | 0.25 ± 0.01 | 0.07 ± 0.02 | 0.21 ± 0.01 |
|  |  |  |  |  |  |  |  |  |  |  |  |  |
| *Megathyrsus maximus* (PCK) | 100 | 25.9 ± 0.25 | 0.45 ± 0.03 | 0.06 ± 0.01 | 0.49 ± 0.03 | 0.42 ± 0.00 |  | 21.1 ± 2.1 | 0.66 ± 0.02 | 0.14 ± 0.02 | 0.21 ± 0.02 | 0.38 ± 0.05 |
|  | 200 | 35.9 ± 0.46 | 0.51 ± 0.03 | 0.10 ± 0.01 | 0.40 ± 0.03 | 0.47 ± 0.01 |  | 28.9 ± 3.2 | 0.67 ± 0.03 | 0.18 ± 0.02 | 0.15 ± 0.03 | 0.26 ± 0.04 |
|  | 400 | 46.9 ± 1.04 | 0.53 ± 0.03 | 0.15 ± 0.01 | 0.32 ± 0.04 | 0.57 ± 0.01 |  | 40.4 ± 3.4 | 0.64 ± 0.05 | 0.18 ± 0.01 | 0.18 ± 0.05 | 0.19 ± 0.01 |

Table S 2. Responses of various electron fluxes to increasing irradiance in leaf of control and shade-grown C_3_, C_4_, gymnosperm, fern, and liverwort species measured under the temperature of 28^o^C and high *p*CO_2_ condition (4%). LEF_O2_ is the linear electron flux determined by the gross rate O_2_ evolution, ETR1 is the total electron flux through PSI calculated using experimentally derived *f*­_I_. CEF is the cyclic electron flux around PSI. Values are means ± s.e.

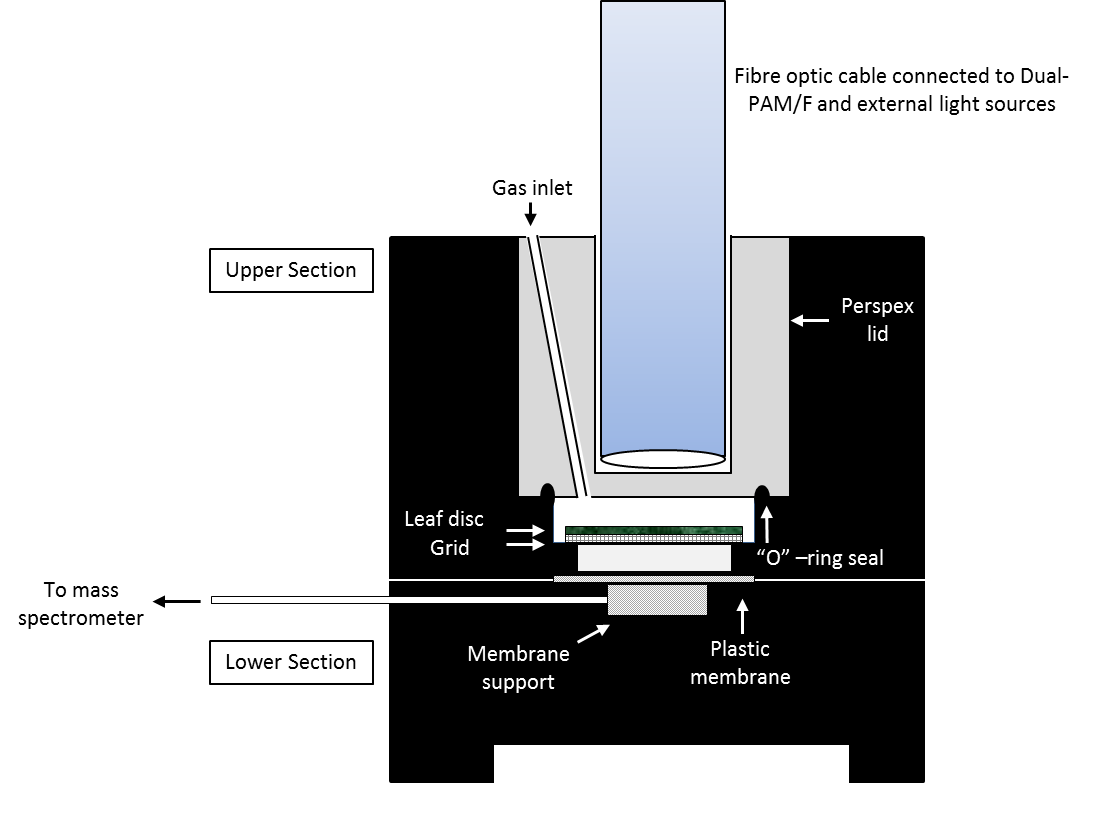


Figure S 1. Cross-sectional diagram of the gas exchange cuvette. The chamber was coupled to a mass spectrometer, Dual-PAM/F, and actinic, strong and weak far-red and saturating light sources as described in the text. The fibre optic cable was housed within the Perspex lid, which together with 2 rubber O-rings created a gas-tight seal. The chamber was linked to the mass spectrometer through a thin, gas-permeable plastic membrane and an ethanol/dry ice water trap. The mass spectrometer (micromass ISOPRIME; Micromass Ltd, Manchester, UK) was operated in peak switching mode for ^18^O_2_ (mass 36), ^16^O_2_ (mass 32) and CO_2_ (mass 44). Net CO_2_ assimilation was calculated from the reduction of CO_2_ concentration, while gross oxygen evolution, gross oxygen uptake and net oxygen evolution were calculated from changes in ^16^O_2_ and ^18^O_2_ respectively, as previously described by Canvin *et al.* (1980).


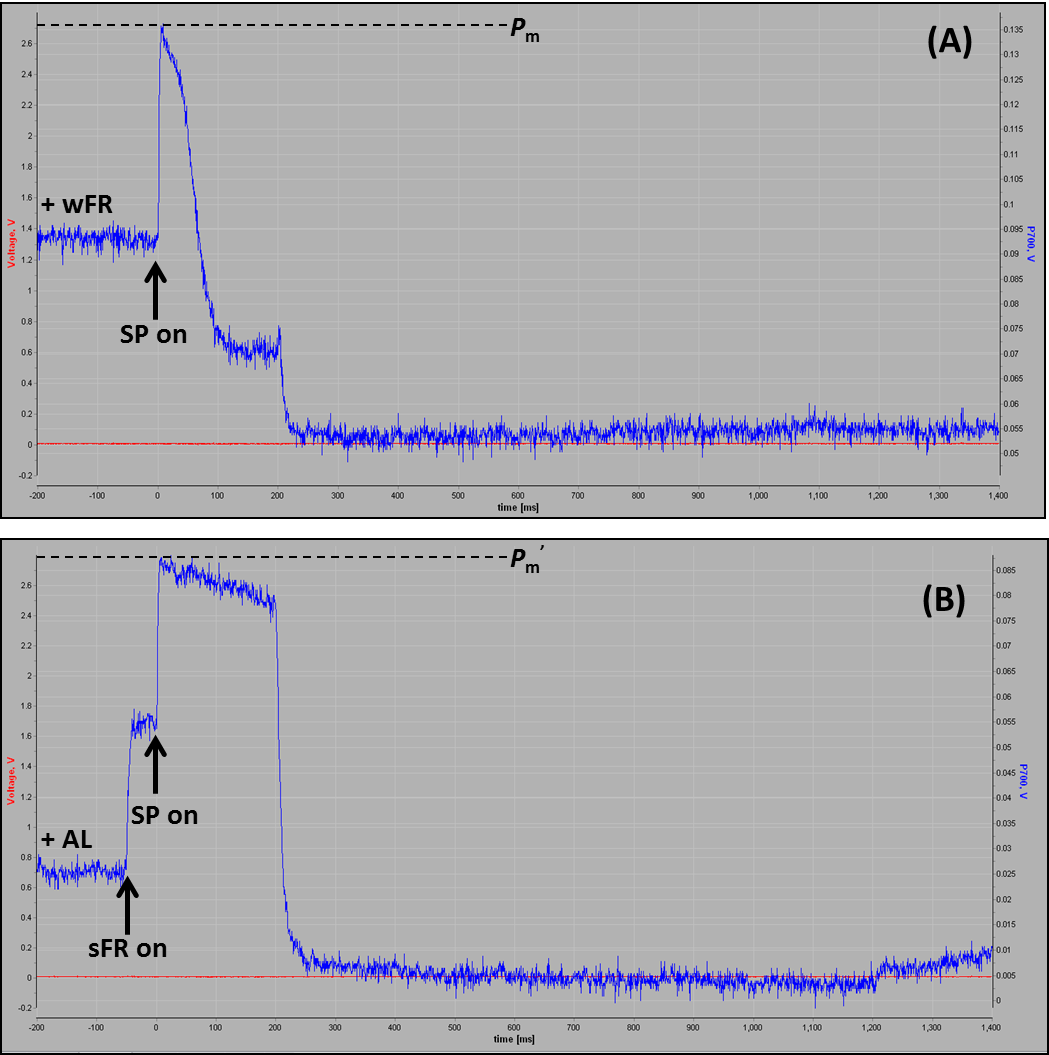


Figure S 2. An example of the determination of energy conversion efficiency in PSI by Saturating Pulse method of Dual-PAM (Klughammer & Schreiber, 2008). (A) The maximum amount of photo- oxidisable P700 obtained through *P*_m_ determination with continuous weak far-red light (wFR) background. (B) Determination of the maximum P700^+^ (*P*_m_’) under actinic white light (AL, 1000 µmol photons m^-2^ s^-1^). The signal relaxed to a baseline corresponding to fully reduced P700.
